# Continuum of sexual and gender-based violence risks among Syrian refugee women and girls in Lebanon

**DOI:** 10.1186/s12905-020-01009-2

**Published:** 2020-08-14

**Authors:** Sophie Roupetz, Stephanie Garbern, Saja Michael, Harveen Bergquist, Heide Glaesmer, Susan A. Bartels

**Affiliations:** 1 Department of Medical Psychology and Medical Sociology, University of Leipzig, 04103 Leipzig, Germany; 2 Department of Emergency Medicine, Alpert Medical School, Brown University, Providence, RI 02912 USA; 3 ABAAD Resource Center for Gender Equality, Beirut, Lebanon; 4 Department of Emergency Medicine, Brigham and Women’s Hospital, Boston, MA 02115 USA; 5 Department of Emergency Medicine, Queen’s University, 76 Stuart Street, Victory 3, Kingston, ON K7L 4V7 Canada; 6 Department of Public Health Sciences, Queen’s University, Kingston, ON K7L 3N6 Canada

**Keywords:** sexual and gender-based violence, Women, Girls, Lebanon, SenseMaker, Refugee, Syria

## Abstract

**Background:**

A myriad of factors including socio-economic hardships impact refugees, with females being additionally exposed to various forms of sexual and gender-based violence (SGBV). The aim of this qualitative analysis was to understand and to provide new insight into the experiences of SGBV among Syrian refugee women and girls in Lebanon.

**Methods:**

The data are gained from a larger mixed-methods study, investigating the experiences of Syrian refugee girls in Lebanon, using an iPad and the data collection tool, SenseMaker®. The SenseMaker survey intentionally did not ask direct questions about experiences of SGBV but instead enabled stories about SGBV to become apparent from a wide range of experiences in the daily lives of Syrian girls. For this analysis, all first-person stories by female respondents about experiences of SGBV were included in a thematic analysis as well as a random selection of male respondents who provided stories about the experiences of Syrian girls in Lebanon.

**Results:**

In total, 70 of the 327 first person stories from female respondents and 42 of the 159 stories shared by male respondents included dialogue on SGBV. While experiences of sexual harassment were mainly reported by women and girls, male respondents were much more likely to talk explicitly about sexual exploitation. Due to different forms of SGBV risks in public, unmarried girls were at high risk of child marriage, whereas married girls more often experienced some form of IPV and/or DV. In abusive relationships, some girls and women continued to face violence as they sought divorces and attempted to flee unhealthy situations.

**Conclusions:**

This study contributes to existing literature by examining SGBV risks and experiences for refugees integrated into their host community, and also by incorporating the perceptions of men. Our findings shed light on the importance of recognizing the impact of SGBV on the family as a whole, in addition to each of the individual members and supports considering the cycle of SGBV not only across the woman’s lifespan but also across generations. Gendered differences in how SGBV was discussed may have implications for the design of future research focused on SGBV.

## Background

### Syrian crisis

Since the start of the conflict in 2011, the situation in Syria has remained one of the worst humanitarian crises in the world [1] with 5,5 million Syrians having fled the country as of June 2020, and another 6 million people internally displaced [2, 3]. Lebanon hosts the largest number of Syrian refugees per capita with approximately one million displaced individuals, and refugees now account for greater than a fifth of the country’s population [2, 4]. Although not a signatory to the 1951 United Nations (UN) Refugee Convention, Lebanon has a long history of hosting refugees including 200,000 Palestinian refugees in addition to 18,500 refugees from Ethiopia, Iraq, Sudan and other countries [5]. As a consequence of the Lebanese government’s decision to not establish formal camps for displaced Syrians, refugees are dispersed across the country, living in informal tented settlements (ITSs) or integrated within local communities, either living with family or in rental accommodations, as opposed to being hosted in formal, supported refugee camps [6, 7]. The highest numbers of Syrian refugees are located in the governorate of Bekaa (339,233) followed by North Lebanon (237,392) and Beirut (215,929) [5]. Nine years into the crisis, Syrian refugees in Lebanon are now more vulnerable than ever, with more than half of them currently living in extreme poverty and over three quarters living below the poverty line [8, 9]. Daily economic challenges significantly affect parents’ ability to adequately care for their children. The increased stress of not earning adequate income to cover the family’s living expenses, can lead to negative mental/physical health consequences, while also contributing to an ongoing cycle of poverty [10]. Having itself experienced war and years of political instability, Lebanon’s precarious infrastructure and resources are significantly strained by hosting more than one million Syrian refugees. This in turn has resulted in tensions within the host communities, which at times has been manifested as violence and discrimination towards Syrian refugees [11]. Displaced Syrian families in Lebanon are also confronted with burdensome governmental policies and regulations. For example, those registered with UNHCR must pledge that they will not work in Lebanon, while other Syrians are only permitted to work in agriculture, construction and environment. Although the Lebanese Government agreed in 2016 to ease work restrictions for Syrian refugees, discussions are still ongoing around implementation of these procedural changes [12–14].

### Sexual and gender-based Violence (SGBV) in humanitarian settings

Lebanon currently hosts about 500,000 Syrian children between the ages of 3 and 18 [9] and it is estimated that 54% of the Syrian refugee population in Lebanon is under the age of 18. While many of these children face risks related to poverty, food insecurity, lack of access to healthcare, and forced labor, girls are also vulnerable to additional gendered risks including child marriage, domestic violence (DV) and intimate partner violence (IPV), sexual exploitation and assault, as well as intimidation and fear of violence within their communities [15–17]. In humanitarian crises women and girls are at risk of various forms of sexual and gender-based violence (SGBV) [18]. SGBV is defined as “any act that results in harm, suffering or humiliation to women and girls and includes rape and sexual abuse, forced pregnancy, dowry-related abuses, female infanticide and domestic violence” [19]. Although a violation of human rights, SGBV has remained widespread with at least one in every three women being beaten, coerced into sex, or otherwise abused in her lifetime [20, 21]. In conflict settings, women are at an even higher risk of SGBV [22, 23] with different manifestations of it sometimes being used as a “weapon of war” to conduct ethnic cleansing [24, 25] and to terrorize people as a method of systematically destroying communities [26]. Mass rape in war has been documented in crises regions such as Bosnia, Somalia, Uganda, the Democratic Republic of Congo and Myanmar [24, 27, 28]. As of today, Rohingyan women in Myanmar and Yezidi women in Iraq face systematic campaigns of sexual violence based on their ethnicity and religious affiliations [24, 29]. At the time of displacement, refugees are also recognized to be at particular risk of sexual victimization with many women being forced into “commercial sex work” or subjected to sexual exploitation and/or torture to pay for their migration [30]. In post-migration settings, women frequently experience social and cultural isolation as well as dire economic need, making them again vulnerable to sexual abuse and exploitation as well as commercial sex work [31]. While SGBV against women and girls during the Syrian conflict, mainly perpetrated by armed actors, is well documented [32], data about SGBV in Syria before the crisis is lacking as it was rarely discussed [33].

### SGBV and gender norms in pre-conflict Syria versus post-displacement

Prior to the war, Syria was very patriarchal and men dominated almost all aspects of society [34]. According to the feminist perspective, men dominated the public sphere whereas women were essentially relegated to the private sphere. This public/private dichotomy served to entrench the patriarchal system and ensured the oppression of women. For instance, women found it difficult to raise issues that impacted them and the political process was biased towards the public sphere whilst largely ignoring the private realm. By marginalising the private sphere, men maintained their dominance of the political process from one generation to the next [35, 36]. Earlier evidence has suggested that gender norms are changing due to the Syrian crisis and its resultant displacement. For instance, Syrian women report feelings more empowered and describe having increased responsibilities with regards to work outside the home and providing for their families [37]. These altered gender norms have been accompanied, however, by increasing reports of SGBV perpetrated by men who use violence as a coping mechanism and to reassert hegemonic patriarchal roles [16]. Under these circumstances, men’s low self-esteem and feelings of disempowerment have contributed to violence against the family as some men have felt stripped of their function in society [34].

Increased rates of child marriage within Syrian refugee populations have been identified [38–40] and studies in Lebanon have highlighted that financial hardships, lack of educational opportunities and safety concerns are the main drivers of early marriage among Syrian refugee populations [38, 39]. Sexual exploitation, forced prostitution and, “survival sex” have also been described as a negative coping strategies for some Syrian women and girls who have no other means to raise the funds needed to cover living expenses in Lebanon [41, 42]. Economic vulnerability also puts many girls and women at risk of sex trafficking [42]. In addition, Syrian women and girls in Lebanon are exposed to physical and verbal abuse, sexual harassment, robbery and fear of being kidnapped [16], often resulting in parents’ reluctance to permit their daughters to leave the house unaccompanied [43].

While many SGBV risks have been well documented and are common across a variety of settings, less is known about the SGBV threats faced by displaced women and girls who are largely integrated into their host communities rather than hosted in formal refugee camps. It is also less common that research on SGBV includes the perspectives of men and boys in addition to that of women and girls. Therefore, what the current study contributes is two-fold: i) an analysis of threats and experiences of SGBV among Syrian refugee women and girls who are integrated into their host communities; and ii) a perspective of SGBV that is broader and more nuanced because it also includes insights from male community members. The ultimate goal of the research is to reduce SGBV risks and to improve assistance to those who have been affected.

## Methods

### Study design and participants

The study was conducted by Queen’s University in collaboration with the ABAAD Resource Center for Gender Equality. Data was collected across three locations in Lebanon hosting a majority of Syrian refugees: Beirut, Beqaa and Tripoli. Twelve interviewers were selected by the ABAAD Resource Center for Gender Equality and completed a four-day training seminar immediately ahead of data collection. The team included six Syrian female interviewers to collect the perspectives of women and girls, as well as three Syrian and three Lebanese male interviewers to collect the perspectives of Syrian and Lebanese men respectively. To represent a broad spectrum of perspectives on the lives of Syrian girls in Lebanon, a convenience sample within each of the following participant subgroups was recruited: married and unmarried Syrian girls, Syrian, Lebanese or Palestinian men, Syrian parents, and community leaders. Within each location, research assistants approached potential participants from their naturalistic community settings including: markets, post offices, commercial settings, bus stops, etc. Only participants aged 13 years and older were eligible to participate in the study. Narratives were audio recorded in Arabic and then transcribed and translated from Arabic into English by native Arabic speakers. Because the story prompts were open-ended, participants could share narratives about any experience of Syrian girls in Lebanon and thus, multiple themes arose from the data. The research presented here is a qualitative analysis of the subset of narratives about experiences of SGBV among Syrian women and girls. All first-person stories by female participants from married and unmarried Syrian girls, Syrian mothers and female community leaders were screened and those about experiences of SGBV were included for analysis (*n* = 327). To ensure that male perspectives were also represented, every fifth story out of 701 stories from male participants was also screened and those about experiences of SGBV were included for analysis.

### Instrument

The dataset is derived from a larger mixed-methods study, investigating the experiences of Syrian refugee women and girls in Lebanon [38]. For our purposes, Syrian girls referred to Syrian females under the age of 18, and this was clearly defined for all participants at the beginning of the survey. In the original 2016 study, data were collected using SenseMaker®, a mixed-method data collection software tool that extracts meaning from a repertoire of stories shared about people’s experiences. SenseMaker was developed by Cognitive Edge and has been used historically for understanding corporate culture, corporate restructuring, etc. Using SenseMaker, participants selected one of three open-ended prompting questions to share an anonymous story about the experiences of Syrian girls in Lebanon: 1. Tell a story about a situation that you heard about or experienced that illustrates the best or worse thing about the life of a Syrian girl (under the age of 18) in Lebanon, 2. Provide a story that illustrates the biggest difference between life for Syrian girls (under the age of 18) living in Lebanon in comparison to life for Syrian girls in Syria, 3. Suppose a family is coming to Lebanon from Syria, and the family has girls under the age of 18. Tell a story about a Syrian girl in Lebanon that the family can learn from. After telling a story, participants interpret their shared narrative by responding to predefined questions on handheld tablets, smartphones or computers. SenseMaker then quantifies each of the responses, providing statistical data backed up by the explanatory narrative [44]. The SenseMaker survey intentionally did not ask direct questions about experiences of SGBV but instead enabled stories about SGBV to become apparent from a wide range of Syrian girls’ lived experiences. In total, 1422 self-interpreted stories from 1346 unique respondents were collected. More detailed information on the survey design and data collection procedure has been previously published [45].

### Analysis

Two researchers (SR, SG) independently screened all 327 first person female narratives as well as the 159 systematically selected male narratives to identify main themes and subcategories around SGBV. Please see below for the definition of SGBV used for screening and coding purposes. Screening discrepancies between researchers were reviewed by a third researcher (HB) and inclusion of the narrative was decided by consensus agreement between all three researchers. Thematic analysis was considered appropriate as it allows rich and detailed meaning to be drawn from data through the emergence of patterns [46, 47]. Analysis consisted of familiarization with the data by three researchers (SR, SG, HB) through reading all narratives multiple times, followed by independent coding to create the set of initial codes. Initial codes were then grouped into potential themes representative of the data by researchers SR and SG. Themes were reviewed by researchers SR and SG and final themes decided by consensus agreement after discussion between researchers SR, SG, HB and SB.

### Definition of SGBV and its use within the study context

For the purpose of this study, stories that discussed any form of physical, sexual, psychological or verbal violence perpetrated against women and girls and seen as a direct result of their gender were screened in and later coded as being about SGBV. Additionally, harassment based on gender, domestic and intimate partner violence of a physical, sexual or emotional nature as well as sexual assault, commercial sex work and exploitation were all included as forms of SGBV. While men and boys are also affected by SGBV, because this study focused on the experiences of Syrian girls, SGBV perpetrated against men and boys was not captured in the current dataset. While early, forced and child marriage is widely recognized as a form of SGBV, these narratives were only included in the current analysis if they also described one of the above forms of SGBV since a separate analysis of early, forced and child marriage from this data has been previously published [38].

## Results

In total, 70 of the 327 (21,4%) first person narratives shared by females discussed some form of SGBV while 42 of the 159 (26,4%) male stories included dialogue on some form of SGBV. Demographics of study participants are presented in Table 1.

**Table 1:** Characteristics of participants who shared stories about SGBV

**Characteristics**	**Female No (%)**	**Male No (%)**
**Gender**	70 (21,4%)	42 (26,4%)
**Subgroup**		
Married Syrian girls	30 (42,9)	–
Unmarried Syrian girls	31 (44,3)	–
Syrian mothers	9 (12,9)	–
Syrian fathers		11 (26,2)
Married Syrian, Palestinian and Lebanese men	–	12 (28,6)
Unmarried Syrian, Palestinian and Lebanese men	–	16 (38,1)
Community leaders	–	3 (7,1)
**Age**		
13–17	48 (68,6)	–
18–24	15 (21,4)	9 (21,4)
25–34	4 (5,7)	21 (50)
35–44	3 (4,3)	6 (14,3)
> 45	–	6 (14,3)
**Marital status**		
Married	28 (40,0)	25 (59,5)
Single	30 (42,9)	17 (40,5)
Divorced/separated	11 (15,7)	–
Prefer not to say	1 (1,4)	–
**Religion**		
Sunni	69 (98,6)	27 (64,3)
Shia	–	3 (7,1)
Other	1 (1,4)	12 (28,6)
**Home region**		
Lebanon	–	21 (50,0)
Homs	–	6 (14,3)
Damascus	11 (15,7)	6 (14,3)
Aleppo	13 (18,6)	1 (2,4)
Hama	10 (14,3)	1 (2,4)
Idlib	6 (8,6)	1 (2,4)
Other	30 (42,8)	6 (14,2)
**Location in Lebanon**		
Beqaa	37 (52,9)	20 (47,6)
Greater Beirut area	22 (31,4)	17 (40,5)
Tripoli	11 (15,7)	5 (11,9)
**Time in Lebanon (years)**		
< 1 year	8 (11,4)	1 (2,4)
1–3	18 (25,7)	5 (12,0)
3–5	37 (52,9)	11 (26,1)
5–7	2 (2,9)	2 (4,7)
> 7 years	5 (7,1)	23 (54,8)

n, number of shared stories

### Qualitative data analysis

Two main themes around SGBV among Syrian refugee women and girls in Lebanon were highlighted in this analysis: 1. Continuum of SGBV risks in the public and private spheres; 2. Gendered differences and perspectives around SGBV.

### Theme 1: continuum of SGBV risks for Syrian women and girls in Lebanon

Different threats and experiences of SGBV in both the private and public spheres were evident for Syrian women and girls in a continuum across the adolescent and early adult years. By private sphere, we refer to acts of violence that occur in the home, typically behind closed doors and often perpetrated by either a family member, friend, or someone else known to the woman or girl. By public sphere, we refer to those acts of SGBV that occur outside the home, in public areas such as schools, streets, shopping areas, parks, etc., and more likely involving an unknown perpetrator. The continuum described in our analysis refers to life stages from unmarried adolescent girls living in the family home, to child brides living with her husband and/or parents-in-law, and in some cases to being divorced or separated after a marriage ends. From this chronological perspective, early SGBV risks refer to those primarily affecting unmarried adolescent girls, while later SGBV risks refer to those experienced more so after being married and leaving the family home.

As adolescents, girls are perceived to be at high risk of sexual harassment and sexual violence in their communities. In an effort to protect them from these public sphere threats and to protect their honor, some parents choose to marry their daughters earlier than they perhaps would have under different circumstances. Child marriage is itself a form of SGBV but the risks are further compounded by the IPV that many girls experience after they are married. In addition to IPV within their marriages, some girls also describe DV by their husbands’ relatives and in a few cases girls were subjected to abuse after they fled violent relationships and returned to their parents’ home.

#### Public SGBV risks for women and girls: harassment and assault

Several forms of SGBV were reported by women and girls as part of their experiences in the public sphere. A majority of the first-person female narratives about SGBV referred to various forms of harassment directed towards women and girls in public spaces, and street harassment was often mentioned as the reason why girls’ mobility was forcibly limited. Harassment was most commonly reported by girls while travelling to and from school as well as in school, creating a sense of unease and fear among some participants. Fear of harassment and heightened concerns around safety for girls led some parents to engage in negative coping mechanisms in an attempt to protect them. For instance, an unmarried Syrian girl based in Tripoli (age 18–24) reported that she was not allowed to attend school.

*“ … When our family moved to Lebanon, I lived in a very unsafe neighborhood, so I was not allowed to leave the house to attend school, fearing that the Lebanese men would take sexual advantage of me or subject me to verbal violence and threats. Moreover, my sister and I are not allowed to stay at home by ourselves because our parents fear that someone might come in and harass us.”*

Some participants described being bullied and harassed not only by boys, but also by teachers and school officials. An unmarried Syrian girl living in Beirut (age 13–17) reported being harassed by her teacher.

*“...One time in class the teacher would ask me to sit right in between two guys, and I would ask him whether he would be ok with his wife sitting in between my father and uncle and he would say “no”. I told him that he has to move me from between the two boys or I would go home, and he would ask me to go home. The teacher kept going on with his hitting and making me sit between guys and I told him that if my father was here he would have never treated me like that … ”*

Living in ITSs or integrated into the host community, many respondents felt unsafe due to experiences and / or perceived threats of sexual harassment and sexual assault on the streets. One Syrian mother based in Beqaa (age 25–34) shared her personal story about being sexually harassed by a male driver while she was walking with her daughter, and discussed concerns for her daughter’s safety in Lebanon.

*“My daughter and I were going to the pharmacy once, when a man in a car started following us and honking. My daughter and I ignored him completely, so he stopped the car and opened the window, only to find him naked in the car. I was very scared for my daughter. He was a disgusting man and what he did was even more disgusting. If the man had done this while she was alone, the man could have kidnapped her. I advise every girl to leave the house with an adult, someone who can protect her.”*

Some female respondents reported being followed and verbally harassed on the streets by passing drivers or while commuting on public transportation. A 22-year old married Syrian girl from Beirut talked about her experiences of harassment and commented that all women were at risk of harassment.

*“When I go to Beirut, the taxi drivers would harass and catcall any women passing by. Regardless if the woman was with her child, married, or pregnant. Some of them would say some very dirty words. When she is walking with a man, they wouldn’t dare to say anything. But they will harass a woman walking alone a lot.”*

One married 18-year-old Syrian girl, who had been in Beqaa for 5 years ago reported being sexually assaulted in the public sphere and discussed misconception and the disrespect she faces as a refugee girl in Lebanon.

*“ … I worked in a chicken restaurant, they disrespected me and assaulted me. The owner was paralyzed and harassed me sexually, he always tried to get close to me. He was old. They think that the Syrian girl has no dignity. I felt really offended and we argued a lot. He gave me a wrong idea of the Lebanese men, they just want us sexually, they think that we sell our dignity for cheap.”*

An unmarried 13-year old Syrian girl living in Beqaa blamed herself for being sexually assaulted on the street and felt that she could not tell her parents about the incident.

*“After some time alone, the boy cornered me in a high place, and then he started holding my hand, touching me in places, and getting his body close to mine. He then wanted to take me somewhere, but I refused, so I wanted to break free but he wouldn’t let me. He kept pulling me towards him, making our bodies collide and touch. After freeing myself from his grip, I ran home and I have not been outside the house since. I know that it is my fault and I am responsible for such action. I cannot tell my parents about this because they will make a big deal out of this. They will blame us and get away with it because we are Syrians. I advise every girl to never play with boys and to never leave the house by herself or without an adult.”*

One married Syrian father living in Beqaa valley (age 25–34) shared a story about a Syrian refugee girl who had lost all her family back in Syria and needed to work to finance herself in Lebanon where she has been sexually assaulted at her workplace.

*“ … She had to work here to support herself so she started by selling flowers. As the days passed she started staying out late for her job. One day, a group of men promised her money if she’d walk with them. They assaulted and raped her. From that day on, the barrier that was once there in her mind was gone so she started working as a prostitute. The girl is a minor, she is 16 years old.”*

Moreover, given the dire financial situation, some women and girls worked in order to support their family’s finances. The employment market in Lebanon, however, often restricts women’s labor opportunities to domestic house work, posing an increased risk of SGBV due to the lack of accountability and reporting mechanisms. Some female respondents reported unwanted sexual advances by their supervisors and in some cases, employers were benefiting from the financial dependency of female employees. For instance, one married Syrian man living in Beqaa (age 25–34) described how an 18-year old girl was harassed by her employers.

*“I know a girl who came to Lebanon with her husband. While they were entering Lebanon, they took her husband on the border. So, she had to work in houses here. Some people used to pay her and others didn’t. She has a little boy who she needs to secure food for. Some people used to harass her while working. So, she never returned to these people’s houses. She is only working for the sake of her son. And she doesn’t know what to do.”*

#### Private SGBV risks: child, early and forced marriage

SGBV risks for Syrian refugee girls were described as one of the main drivers for parents to marry their daughters early, since marriage and having a husband were perceived as protective against SGBV. One 16-year-old married Syrian girl in Beqaa was married at the age of 14 as a protection measure due to safety concerns.

*“ … I got married because my parents feared for my safety. There were a lot of kidnapping cases happening in our village. … I have marital problems as well. I advise parents to reject the idea of early marriage. It is not right. The girl should be allowed to live through her whole childhood. I will not approve for any of my daughters to get married at an early age; especially after this experience that I am going through.”*

This Syrian girl in Beqaa chose to get married at the age of 14 and gave up education due to a fear of being harassed and assaulted.

*“When I was 14 years old, I chose to get married. This was due to my former school principal; he used to harass the students and teachers. He constantly harassed them. After a while, he started to harass me. Therefore, I hated school, and I accepted to get married to the first man who proposed to marry me. I was relieved after I got married. I was relieved from men’s harassment. I preferred to get married. Now, I am very comfortable. I hated being harassed. I saw how the teachers harassed the girls at school. … More than one girl from my school decided to get married. Even though I would have preferred to continue my education, I was compelled to marry in order to get rid of the harassment.”*

One Syrian girl who was displaced with her parents to Beirut at the age of 12 was forced into marriage to protect her from further harassment after being targeted by a man in the community.

*“ … In Lebanon, I was sexually harassed from an old aged man. When my parents knew, they forced me to get married. Now, I have a child, and I am pregnant. I am unhappy.”*

Early, child and forced marriage is a form of SGBV because it forces girls to have sex before they are old enough to consent and before their bodies are physically mature. In some cases, the outcomes are extreme as in this example shared by a married Lebanese man in Beirut (age 45–54).

*“...My daughter is only 9 or 10 years old. The employer sees my daughter, and he asks to either marry her or to buy her. I accept since I don’t have any other solution, but my wife starts shouting and crying. We got a clerk and married the employer to our child, and he took her as his wife … Our daughter died the next day from an extensive hemorrhage that was the result of brutal intercourse. He is a monster. Our child died. This is a true and very sad story. I hope, by documenting and sharing this story, to spread awareness to the public.”*

#### Additional private SGBV risks: intimate partner violence and domestic violence within marriages

In addition to experiencing early marriage as a form of SGBV, married Syrian girls were sometimes exposed to violence in their homes after being married at a young age. In the private sphere, intimate partner violence (IPV) against women and girls in their own homes was commonly reported. Other girls experienced domestic violence (DV) perpetrated by other family members, while a few girls were sexually exploited by their parents, often under the guise of marriage. IPV was a prominent form of SGBV discussed by respondents in this analysis. Much of the narratives around IPV were linked to a power differential since the bride was a child/minor and/or was forced into a marriage. For instance, one 15-year old married Syrian girl (age 13–17) in Beqaa reported on the abuse she experienced by her husband.

*“ … I got married to another man, and my husband abuses me. I have two daughters now. I wouldn’t have gotten married, if I knew what I was going to go through. I am not happy … I am 15 years only. I lost my life. I advise girls not get married before the age of 20.”*

This Syrian girl located in Tripoli (age 18–24) reported being married at the age of 18 and described her experiences of violence.

*“My husband has anger issues, and he rarely works. I used to have a job, but he prohibited me from working. When he gets mad, he starts swearing at me and beating me. I live with my parents-in-law. I like living with his parents because I don’t like my husband.”*

Overall, physical violence perpetrated by husbands was the most commonly reported form of IPV. A Syrian girl based in Tripoli (age 13–17) reported physical abuse by her husband during their brief marriage.

*“I was 15 years old when I got married. I didn’t get engaged, and I didn’t do a wedding. We were married in four days only. Problems started immediately. I lived with him for 20 days only. He used to beat me, he wanted to send me to Turkey, he wanted me to work as a dancer, and he wanted to take me away from my parents. One day, I ran away from him. People I know helped me, to get from where he lived to my parent’s village. Then, my parents helped me to get a divorce.”*

Another Syrian girl from Beqaa (age 18–24) was forced into marriage at the age of 16 and then experienced IPV from her husband.

*“At first, I refused then my parents encouraged me. I didn’t love my husband. He prohibited me from visiting my parents, and he beat me. At first, we lived with his parents then his father kicked us out. We moved a lot. My parents didn’t stand by my side. I suffered a lot. I tried to kill myself.”*

This Syrian girl (age 13–17) in Tripoli chose to marry her husband; at first the marriage was happy but then the girl experienced IPV when she was beaten by her husband and DV when she was beaten by her brother- in-law.

*“ … My husband and my brother-in-law started to beat me. I started to yell, so our neighbor came to help me. But they kept on beating me. My mom picked me up and took me to the doctor. We got a report, and we hired a lawyer to get my son back. Now, my son is with me, and I returned to my parent’s house waiting to get divorced.”*

Another married Syrian girl in Beqaa (age 13–17) was similarly exposed to both IPV and DV after being pressured into marriage.

*“One of my father’s relatives wanted to marry me. At first, I didn’t accept, but then they convinced me that he is a good man, and I accepted. We fought constantly. Then I fought with his parents. My husband, his mother, and his uncle beat me. Even their guests would beat me. I suffered a lot when I lived with them. I neither ate nor drank anything, and I always stayed alone.”*

#### SGBV risks after leaving unsafe marriages: domestic violence and sexual exploitation

Despite finding themselves in unsafe and unhealthy marriages, some Syrian girls continued to face violence and abuse even as they sought divorces and attempted to flee abusive situations. Due to traditional values, social stigma and taboo surrounding divorce, this violence was often perpetrated by the husband’s and / or the girl’s family and there was a perception that the girls were blamed for their unsuccessful marriages. For instance, one married Syrian girl in Beqaa (age 13–17) was maltreated by her parents after fleeing her abusive husband.

*“They [husband and his family] would yell at me and insult me all the time. I stayed there for 20 days, and I couldn’t take it anymore. I returned to my parents’ house, and they told me that everything will be okay after my husband and I reconcile. When I told them I do not wish to reconcile things with my husband, they started to beat me as well. They took me to a clerk (Sheikh) to check if I was possessed or something. I suffered at my parents’ house and at my parents-in-law’s house. I cried all the time.”*

As a result of the dire economic need, some girls were sexually exploited by their parents under the guise of marriages. For instance, this Syrian father (age 25–34) from Beqaa shared a story about a Syrian family that was displaced to Lebanon with their 12-year old daughter.

*“The father married his daughter to a Lebanese man. This man got married to her because he wanted to have fun. After a while, he divorced her. Her parents married her to a Syrian man. Her father has married her seven times, and he would increase her dowry every time; he is benefiting from her dowry. This girl is younger than 18 years old, and she doesn’t know what is going on. Her story is known all over the Syrian camps.”*

As a result of being refused by relatives after a divorce due to shame and dishonor, some girls turned to commercial sex work to support themselves. One Lebanese man in Beqaa (age 25–34) shared a story about a 17-year old Syrian girl who tried to return to her parent’s home after being physically abused by her husband.

*“They got her married to a Lebanese guy. After a while of their marriage, which lasted less than a month (25 days), the guy and his family started hitting her, she ran away to her parents’ house, but they did not welcome her because she is a married woman now. However, the neighbors pressured her parents to let her into the house, but after 3 days the girl disappeared. A neighbor they know saw her in Beqaa, but she acted as if she didn’t know or see him. And he finds out that she is working in prostitution; her parents are trying to get her back now.”*

### Theme 2: gendered differences discussing SGBV risks

SGBV risks for female Syrian refugees were discussed differently by female and male respondents. For instance, experiences and fears of sexual harassment were mainly reported by women and girls, with 48 of 70 female narratives presenting sexual harassment as the primary form of SGBV in Lebanon. There were no accounts of sexual exploitation shared by women or girls in this study.

This unmarried Syrian girl in Beqaa and her school friends were harassed while waiting for the bus (age 13–17).

*“ … My friends and I, who are waiting for the bus, are all girls and there were children amongst us as well. During the walk, a car with black tinted windows started following us; every now and then it would shine the front lights and honk. When we reached our camp, the car drove off. My friends and I were terrified.”*

Another unmarried Syrian girl in Beqaa (age 13–17) reported how unsafe she felt as a girl in Lebanon due to harassment, comparing her situation in Lebanon to that in Syria.

*“ … We are living in tents and I cannot go out and walk around. I can’t even smell the fresh air (the fresh air has been prohibited for me). I am not even sad that I cannot go out in the city because there are young men that sit outside and I am scared to go outside because they bother us and I am a girl, I do not want to go out.”*

In contrast, only 8 of 42 male respondents discussed sexual harassment against Syrian girls. Instead men were much more likely to talk explicitly about sexual assault (14 out of 42) and sexual exploitation (19 out of 42). For instance, this unmarried Lebanese man from Beirut (age 18–24) reported a story on sexual abuse where one man wanted to marry a Syrian girl, but her father refused.

*“The guy started threatening to kill her father if she does not marry him. Every day, a different man would sexually abuse her until the girl, 17 years old by that time, broke down mentally and she started talking to herself, prompting everyone to call her crazy. Furthermore, this man and his group of friends surrounded the house, banning anyone from coming in or out. Her father tried leaving the house once, but they would not let him. The girl contemplated suicide because of what she has suffered.”*

One Syrian man (age 25–34) in Beqaa shared a story about a 16-year old girl who was forced into commercial sex work to financially support her family.

“*I know a Syrian family that displaced to Lebanon. They have three daughters. When one of their daughters was 16 years old, she was compelled to work in sinful routes due to the family’s circumstances. She got raped, and she worked in prostitution. Now, she is a drug addict, and she is 18 years old.”*

In some cases, the dire economic need led to coerced commercial sex work. For instance, a married Syrian man located in Beqaa (age 25–34) shared a story about a 17-year old Syrian girl who was forced into commercial sex work in order to pay for rent:

*“They came here after the war. She lived with someone here in this area. He abused her age and bad situation and made her work in prostitution. It started as a secret, but then we found out what was happening with this girl. He is abusing her in return of a little amount of money, let’s say for rent maybe.”*

Similarly, a Syrian father in Beqaa (age 25–34) shared a story about Syrian girls who were promised job opportunities but ended up as commercial sex workers.

*“I know three girls who came from Damascus. They are students at school; the youngest one is 18 years old to 16-17 years. They are under the age of 20. They came to work at restaurants and cafes, but they used them and took them to prostitution places, for the sake of money. Several girls came, not only one.”*

Sexual exploitation in the form of short term, contractual marriages was also discussed as illustrated by this Syrian father from Beqaa (age 35–44).

*“ … She is getting married for two weeks or a month, and then she’ll get divorced. I asked one of the girls how is she? She told me that she resents herself. She is being exploited. They are continuously getting married, and they are still minors. Even in her few weeks with one of her husbands, she’ll be abused. … I know a girl who has been married 6 times. This is wrong.”*

## Discussion

In this analysis, SGBV was identified as an important issue by both women and men, even though the study design and methodology did not include any prompting about the topic, neither in the study introduction nor in the questions. Given the methodology, experiences and threats of SGBV arise naturally from a broad spectrum of lived experiences. Sexual harassment in the public sphere was the most common form of SGBV identified in this study, followed by sexual abuse and exploitation, which is consistent with previous research [16, 17, 48]. Despite facing a range of SGBV threats, Syrian women and girls also demonstrated their agency by making strategic decisions to protect themselves from SGBV threats (for instance, leaving school and leaving abusive relationships). It is important to recognize their strength and resourcefulness despite living with very constrained choices.

In Lebanon, Syrian refugees are largely integrated within their host communities, as opposed to living in formal refugee camps, where SGBV risks have been better documented [6, 7]. Hosting Syrian refugees has strained Lebanon’s resources and infrastructure, which has created tension, and at times hostility, within the host community towards displaced Syrian families [12, 49]. What this analysis adds to the existing literature is a more nuanced understanding of SGBV risks faced by refugees who are integrated into such a host environment. This is especially relevant since most of the perceived SGBV threats for girls were derived from the host community itself (particularly from the perspective of women and girls). For instance, since work permits are restricted in Lebanon [50, 51], some Syrian women and girls are forced to work to meet their families’ basic needs. Since this work is usually in the informal sector, it can increase their risk of sexual abuse and exploitation [52]. Furthermore, since some Syrian women and girls are not registered in Lebanon, they are understandably hesitant to report sexual assault since reporting could lead to detention and deportation [15]. Women and girls also faced SGBV risks in their private lives with significant threats reported from husbands, from the husband’s family, and from the girl’s own family. IPV perpetrated by husbands was the most common form of violence in the private sphere, consistent with other existing evidence [18, 53]. IPV is thought to be exacerbated among displaced Syrian families by the frustration that some men experience when their lives are disrupted, when their pre-existing roles as breadwinners within the family are altered, and when traditional gender norms are challenged [16, 54]. Girls who marry young and have a large age gap with their spouses seem to be more at risk of IPV related to systems of power [39, 55]. Restricted educational opportunities and insecurity increases girls’ dependency on men, thereby limiting their agency and perpetuating patriarchal gender norms [39, 56]. While Syrian men may have lost some of their patriarchal power due to displacement, women and girls are often challenged to take on more responsibilities, both inside and outside the home [37]. These new gender roles, as well as an unsafe environment and severe economic strain may increase stress levels and contribute to men perpetrating IPV. Increased levels of IPV have been reported during other periods of armed conflict in the region included during the second Intifada in the West Bank and Gaza [29] as well as among Palestinian refugee communities in Lebanon [57].

### Continuum of SGBV risks

Our analysis highlights that female Syrian refugees are at heightened risks of SGBV in their host communities. However, the types of SGBV experienced appear to differ across the adolescent and early adult years. As earlier risks of SGBV, sexual harassment and sexual assault were experienced in the public sphere limiting girls’ mobility as well as access to education and work. It has been documented that if a girl experiences sexual abuse, her risk of subsequent sexual and physical victimization is significantly increased [58] and this phenomenon was evident in the current research. As a result of shame and dishonor, these SGBV threats and experiences in the public sphere lead to other SGBV risks in the private sphere. For instance, unmarried Syrian girls who are perceived to be at risk of sexual harassment and sexual assault in the community are sometimes pressured or forced into child marriage as a means of “protecting” them. Child marriage is itself a form of SGBV since girls are forced to have sex before they are able to give consent, and sometimes before their bodies are physically mature. In essence, some families exchange the threat of sexual harassment or sexual assault in the community for another form of SGBV, namely child marriage, as they believe that it will protect the honor of the girl as well as the honor of the family [59]. The cultural and gender norms that render girls unmarriable and their families dishonourable in the event of sexual harassment [60], sexual assault or pre-marital dating, directly contribute to girls experiencing the next type of SGBV, i.e. child marriage. After their marriages, sometimes to older men with the age inequality further compounding the gender inequality, many child brides face the additional threat of IPV in the form of physical and emotional abuse. Again, this phenomenon has been well documented in previous research [34, 61]. Leaving violent relationships can be challenging particularly for young brides as there is sometimes family and societal pressure to maintain the marriage despite violence. If women attempt to leave their abusive relationships they may then be at risk of abuse by their own families including physical and emotional abuse as well as sexual exploitation, as they are often blamed for their failed marriages [62]. Children born into this cycle of violence are more likely to witness IPV and to experience DV [63], with this exposure increasing the risk that the children will personally experience and perpetrate violence later in life [64–66], thus continuing an intergenerational cycle of violence.

### Gendered differences discussing SGBV risks

Although SGBV was noted to be an important issue for both men and women there was a gendered difference in how SGBV risks were discussed. Female respondents mainly described experiences and/or fears of sexual harassment in the public sphere and talked about the resultant limitations in their daily movement and freedom. Among women and girls, the notable lack of dialogue around IPV and DV aligns with previous portrayals of IPV and DV as private issues. Challenging the privacy of SGBV and attempts to address/report it has sometimes been perceived as a betrayal of family and a violation of social cohesion [67, 68]. In contrast, men more explicitly and openly discussed sexual assault and sexual exploitation. In some cases, male respondents identified commercial sex work as a form of sexual exploitation, often related to the family’s dire economic needs similar to other research [69]. It is noteworthy that there were *no* cases of sexual exploitation mentioned by women or girls despite interviewing men and women from the same communities. This may be secondary to the stereotypic differences in gender socialization whereby women are raised to shy away from talking about sex, but boys are socialized with less taboo around sex [70]. If the analysis had only included narratives from female participants, the more overt forms of SGBV such as sexual exploitation and abuse would not have been appreciated. It is also likely that SenseMaker contributed to this finding since it asks broad, open-ended questions, thus allowing participants to decide what they would like to share. This observation may be relevant and have implications for the design of other SGBV research. Since many SGBV studies focus exclusively on women and girls, they may be missing important findings and our experience in the current work would suggest that researchers should consider additionally including men and boys, particularly if stereotypical taboos around sex are anticipated to prohibit women from sharing their experiences / concerns openly.

### Strategies to address SGBV

The study’s findings demonstrate a varying array of SGBV threats faced by Syrian women and girls in Lebanon, and these threats require a multi-pronged prevention and protection strategy.

#### Consider protection strategies in the context of refugee integration into the host community

First, protection strategies must consider particular SGBV risks arising from the integration of Syrian refugees into host communities, and how inherent tensions likely contribute to feelings of insecurity and perceived SGBV threats. In Lebanon’s ITS, Syrian women and girls have limited access to essential SGBV programs due to lack of transportation, as well as cost and gendered expectations around mobility and domestic responsibilities. Recognizing that Syrian women and girls are a hard-to-reach population, a few organizations have piloted mobile service provision and an initial evaluation of one model showed promising potential to increase refugee’s social networks, knowledge and self-confidence [71]. A replication of such mobile services nationwide may therefore have the potential to reduce barriers to SGBV services. Additionally, these programs should serve as one-stop shops for women and girls where they can access different services all in one place, while also following the GBV Call to Action 2016–2020 in emergencies by providing a minimum package of legal services, case management, and referral.

#### Addressing patriarchal social norms

Second, addressing SGBV must challenge patriarchal social norms and the socialization of men towards the use of violence, in addition to raising awareness about the negative effects of SGBV. Engaging men in SGBV prevention programming is critical to challenging social norms, attitudes and practices that increase the risk of SGBV while also harnessing positive male power to prevent violence and promote safety. Veale et al. [72] found that, through guided discussion on masculinity and gender roles, Syrian men talked about their changed circumstances as refugee men and fathers. In this work, a safe emotional space was created for men to collectively talk about their problems, to become more attuned and reflective about their relationships with their wives and children, and to engage in better emotional regulation. Through dialogue, men started to distance themselves from earlier responses of anger and frustration and became more empathic and reflective on their own needs as well as those of their families [72]. Several other initiatives have been piloted in Lebanon targeting men and boys as a means to alleviate the burden of some forms of SGBV, and in 2014 the Men Engage Lebanon Country Network was launched [73] to harmonize these efforts, while ensuring minimal duplication of interventions. Little evidence-based curricula are currently available to guide front-line staff on how to engage with men and boys and future efforts should prioritise this gap to produce context specific and culturally sensitive tools.

#### Addressing structural drivers of SGBV including legal reform

Lastly, in line with Heise’s ecological model, which conceptualizes SGBV as being perpetuated through multi-layered factors including intra-individual level, microsystem, exosystem and the macrosystem, programming efforts should also address the overarching structural problems that contribute to SGBV [53]. In their adapted social ecological model for GBV against Syrian women in Lebanon, Yasmine and Moughalian [74] argue that interventions designed to focus on the intrapersonal level fall short from effectively influencing a change. They recommended that programming should aim at challenging existing power dynamics and urged organizations to look at structural determinants that contribute to SGBV during forced displacement, such as lack of employment, dire economic situation, as well as security and protection concerns [74]. For instance, Lebanon adopted legislation in 2014 aimed at better protecting women from SGBV, but feminist and women’s right organizations argue that the law falls short of addressing all forms of SGBV and more robust legislation is called for [48, 75] especially when it comes to the protection of Syrian and other refugee populations. Further legal reforms are also necessary including laws against street harassment and work protection laws, as well as modifying the age requirement for marriage to 18 years. Additionally, policy reform to facilitate legal registration and obtaining official documents is urgently needed in Lebanon since women and girls are vulnerable to sexual exploitation and assault, yet currently have little mechanism for recourse without risking detention and deportation.

### Strengths and limitations

This qualitative study has several limitations. First, the sample is not representative of the Syrian refugee population in Lebanon and therefore the results are not generalizable. Girls less than age 13 were excluded and since this study is qualitative in nature there is no consideration of the subgroups’ balance. Second, due to time and human resource constraints we were not able to analyze all male narratives, and this may have also introduced a bias. However, a systematic sampling method was utilized in an effort to be objective. Third, the SenseMaker narratives are often quite brief, and without the follow up questions typically asked in qualitative interviewing, the shared stories sometimes lack the detail and richness expected of in-depth qualitative interviews. Finally, it was sometimes not clear whether the SGBV experiences described were perceived to result from being a Syrian refugee, from being a female or from a combination of the two. The study also has a number of strengths worthy of mention. Since the SenseMaker survey did not ask directly about SBGV, participants shared their stories more naturally as part of their broader experiences. Moreover, the current analysis offers a variety of perspectives from women and girls of different ages as well as from men and it illustrates the various SGBV risks faced by female refugees who are integrated into their host communities rather than housed in formal refugee camps.

## Conclusions

Our findings shed light on the importance of recognizing the impact of SGBV on the family as a whole in addition to each of the individual members, and to also consider the cycle of SGBV not only across the woman’s lifespan but also across generations. Furthermore, this analysis contributes to our understanding about the SGBV risks faced by female refugees integrated into the host community rather than hosted in formal refugee camps, highlighting how SGBV is intimately related to other aspects of social welfare as well as being part of the everyday lives of Syrian girls. Syrian girls are exposed to early SGBV risks in the public such as harassment and assault, which sometimes leads to child marriage as the next form of SGBV experienced. Within early / forced marriages, some girls face additional SGBV risks in the private sphere in the form of IPV and/or DV. In this analysis, female respondents were more likely to share stories about sexual harassment, while male respondents were more likely to discuss explicit forms of SGBV such as sexual exploitation, and this may have implications for the design of future research focused on SGBV prevention and response.

## Data Availability

The datasets used and/or analysed during the current study are available from the corresponding author on reasonable request.

## Abbreviations

DV: Domestic Violence
IPV: Intimate Partner Violence
ITSs: Informal Tented Settlements
SGBV: Sexual and Gender-Based Violence
UN: United Nations
